# Survival Response of Hippocampal Neurons under Low Oxygen Conditions Induced by *Hippophae rhamnoides* is Associated with JAK/STAT Signaling

**DOI:** 10.1371/journal.pone.0087694

**Published:** 2014-02-06

**Authors:** Manimaran Manickam, Rajkumar Tulsawani

**Affiliations:** Defence Institute of Physiology and Allied Sciences, Defence Research and Development Organization, Delhi, India; National University of Singapore, Singapore

## Abstract

Janus activated kinase/signal transducers and activators of transcription (JAK/STATs) pathway are associated with various neuronal functions including cell survival and inflammation. In the present study, it is hypothesized that protective action of aqueous extract of *Hippophae rhamnoides* in hippocampal neurons against hypoxia is mediated via JAK/STATs. Neuronal cells exposed to hypoxia (0.5% O_2_) display higher reactive oxygen species with compromised antioxidant status compared to unexposed control cells. Further, these cells had elevated levels of pro-inflammatory cytokines; tumor necrosis factor α and interleukin 6 and nuclear factor κappa B. Moreover, the expression of JAK1 was found to be highly expressed with phosphorylation of STAT3 and STAT5. Cells treated with JAK1, STAT3 and STAT5 specific inhibitors resulted in more cell death compared to hypoxic cells. Treatment of cells with extract prevented oxidative stress and inflammatory response associated with hypoxia. The extract treated cells had more cell survival than hypoxic cells with induction of JAK1 and STAT5b. Cells treated with extract having suppressed JAK1 or STAT3 or STAT5 expression showed reduced cell viability than the cell treated with extract alone. Overall, the findings from these studies indicate that the aqueous extract of *Hippophae rhamnoides* treatment inhibited hypoxia induced oxidative stress by altering cellular JAK1, STAT3 and STAT5 levels thereby enhancing cellular survival response to hypoxia and provide a basis for possible use of aqueous extract of *Hippophae rhamnoides* in facilitating tolerance to hypoxia.

## Introduction

High altitude illnesses is a term that indicates various symptoms of acute mountain sickness comprising headache, nausea, pulmonary edema and cerebral edema occurring during rapid ascent to high altitude by an un-acclimatized sojourn [1], [2]. Impairment of cognitive functions, memory loss, anxiety and depression are some of the nervous system related ill effects of hypoxia [3]. Neuronal damage during hypoxia is well documented and oxidative stress is known to be a cause [4], [5]. Acclimatization to high altitude environment helps alleviate related disorders and hence finding an agent of herbal origin that promotes acclimatization at a faster rate will be promising to tackle the problems during rapid ascent to high altitude.

Hippocampus neurons are the first ones to lose their electrical activity during hypoxia [6]. Protecting hippocampus from hypoxia will prevent many cognitive disorders. Cellular responses including proliferation and survival to many external signaling molecules are executed via Janus kinase-signal transducers and activators of transcription (JAK/STAT) signalling pathway [7]. Janus kinases (JAKs) comprises of four tyrosine kinases namely, JAK1, JAK2, JAK3 and Tyk2. Signal transducers and activators of transcription (STATs) include seven structurally and functionally related proteins namely, STAT1, 2, 3, 4, 5a, 5b and 6. Cytokine binding with its corresponding receptor initiates the signalling cascade of JAK/STAT pathway [8].

Hypoxia alters the cellular redox state which leads to oxidative stress and neuronal damage. Antioxidant defence system of the cell is weakened during hypoxia with an overload of reactive oxygen species [9]. Endogenous antioxidants like reduced glutathione and superoxide dismutase levels reduce during hypoxia. Pro-inflammatory cytokines such as TNFα (Tumour Necrosis Factor α) and IL6 (Interleukin 6) are markers of inflammation and NFκB (Nuclear Factor kappa B) regulates these pro-inflammatory cytokines at transcriptional level [10]. JAK/STAT signalling is responsible for many cellular events and we hypothesised that hypoxia might have an effect on JAK/STAT signalling to elicit its effects.

*Hippophae rhamnoides* is a high altitude plant, which was once considered as a weed has now proved to have many medicinal properties like antioxidant, anti-inflammatory, cardioprotective activity and cytoprotective activity [11], [12]. *Hippophae rhamnoides* leaves are rich sources of antioxidants and polyphenols [13]. Therefore, it is hypothesized whether the *Hippophae rhamnoides* extract treatment induces JAK/STATs signaling to fine tune TNFα mediated responses thereby providing neuronal survival under hypoxia environment.

## Results

### Chemical Standardization of Sub Critical Aqueous Extract of Hippophae rhamnoides by its Total Phenolics, Flavonoid Content, Antioxidant Activity and Fingerprinting

**Figure 1:**
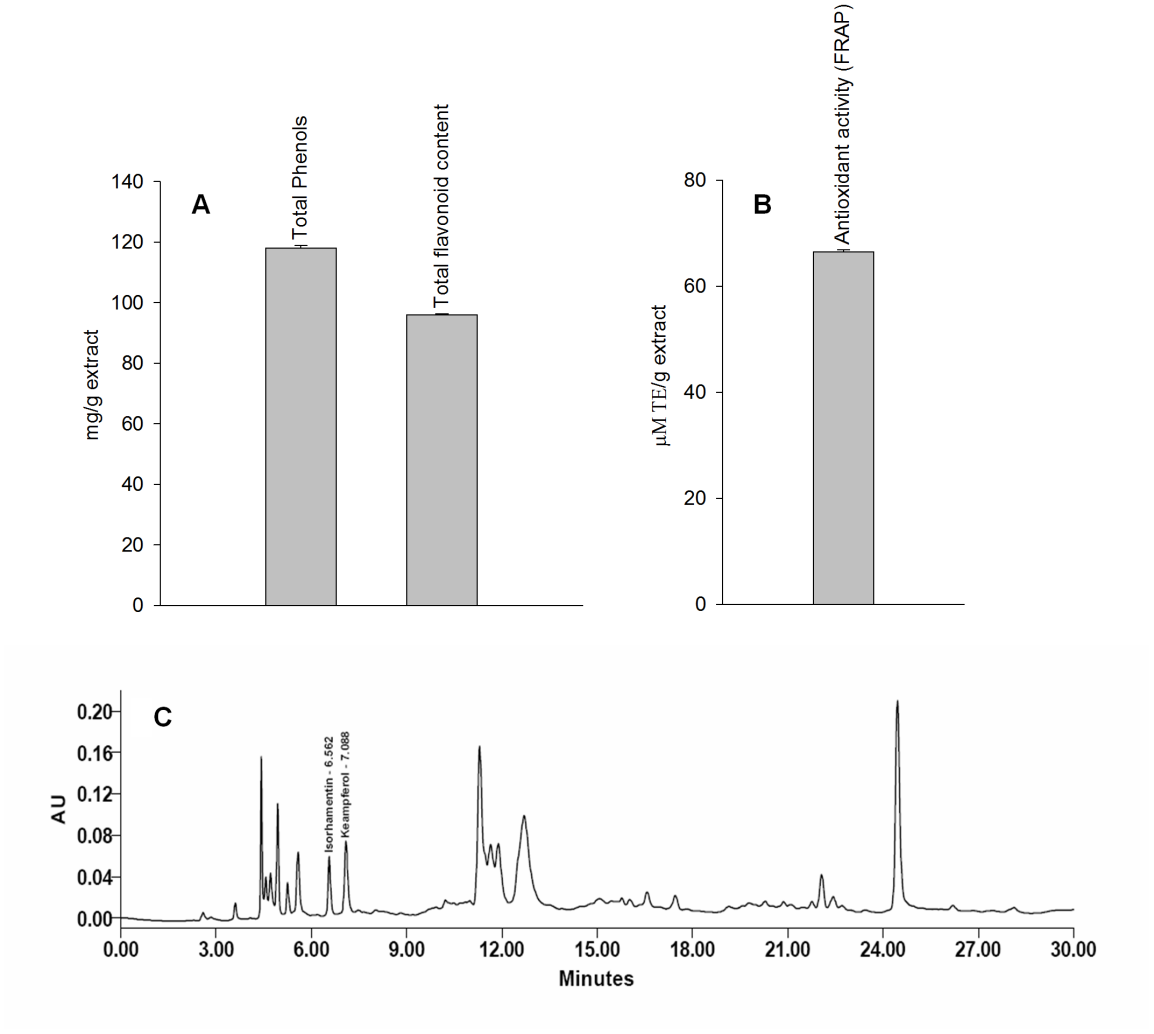
Depicts total phenols and flavonoid content (A), antioxidant activity (B) and RP-HPLC chromatogram (C) of aqueous extract of *Hippophae rhamnoides*.

The % yield of aqueous extract of *Hippophae rhamnoides* ranges 30.0–33.2%. The phenolic and flavonoid content in aqueous extract was 118.0±0.88 mg/g extract and 96.0±0.36 mg/g extract, respectively (figure 1A). The antioxidant activity of extract was found to be 45.0±0.12 µM TE/g extract (figure 1B). HPLC profile of extract showed the presence of isorhamnetin and kaempferol which are chemical markers compounds reported in *Hippophae rhamnoides* and are responsible for many pharmacological effects documented.

### Effect of Hippophae rhamnoides Treatment on Cell Viability, Reactive Oxygen Species, Caspase 3 and Lactate Dehydrogenase Leakage in HT22 Cells Exposed to Hypoxia in the Presence or Absence of Hippophae rhamnoides Extract

**Figure 2:**
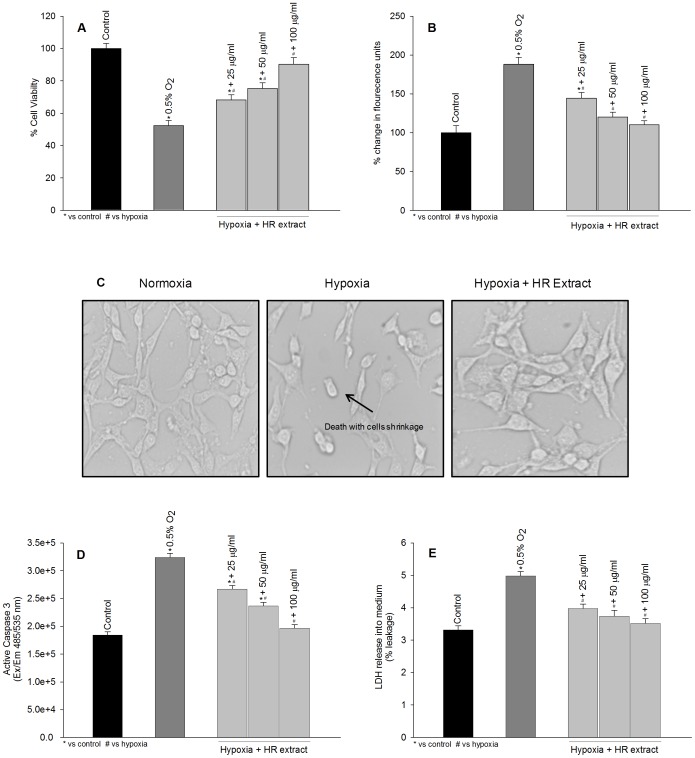
Protective efficacy of *Hippophae rhamnoides* extract on hypoxia induced cell death. (A) effect of extract on hypoxia induced on cell death (B) effect of extract on hypoxia induced reactive oxygen species generation (c) photomicrograph of HT22 cells following hypoxia exposure with or without extract, 40x magnification (d) active caspase 3 expression in HT22 cells following hypoxia exposure with or without extract (e) lactate dehydrogenase leakage in HT22 cells following hypoxia exposure with or without extract. *p<0.05; * vs control; ^#^ vs hypoxia.

Figure 2 depicts protective effects of *Hippophae rhamnoides* extract in HT22 cells exposed to hypoxia for 24 h in the absence or presence of extract. The cells exposed to hypoxia in absence of extract showed significant cell death with higher levels of reactive oxygen species (figure 2A–B; dark gray bars). Treatment of cells with extract showed dose dependent cyto-protection (figure 2A; light gray bars) and prevented increase in reactive oxygen species (figure 2B; light gray bars). The cells exposed to hypoxia showed shrinkage and changes in the morphology of cell which was prevented by the extract treatment (figure 2C). To understand mode of cell death, the levels of active caspase 3, marker for apoptosis and lactate dehydrogenase leakage, a necrotic marker was evaluated. Cells subjected to hypoxia revealed 1.76 fold increase in active caspase 3 and 1.27 fold leakage of lactate dehydrogenase into the cell culture medium (figures 2D–E; dark gray bars). These cells exhibited both apoptotic and necrotic modes of cells death however; the cell death was predominantly apoptotic. Treatment of cells with extract prevented activation of caspase 3 and lactate dehydrogenase leakage induced by the hypoxia and the effects were dose dependent (figures 2D–E, light gray bars). Overall, *Hippophae rhamnoides* treatment attenuated cell death and reactive oxygen species induced by hypoxia.

### Antioxidant Status of HT22 Cells Exposed to Hypoxia in the Absence or Presence of Hippophae rhamnoides Extract

**Figure 3:**
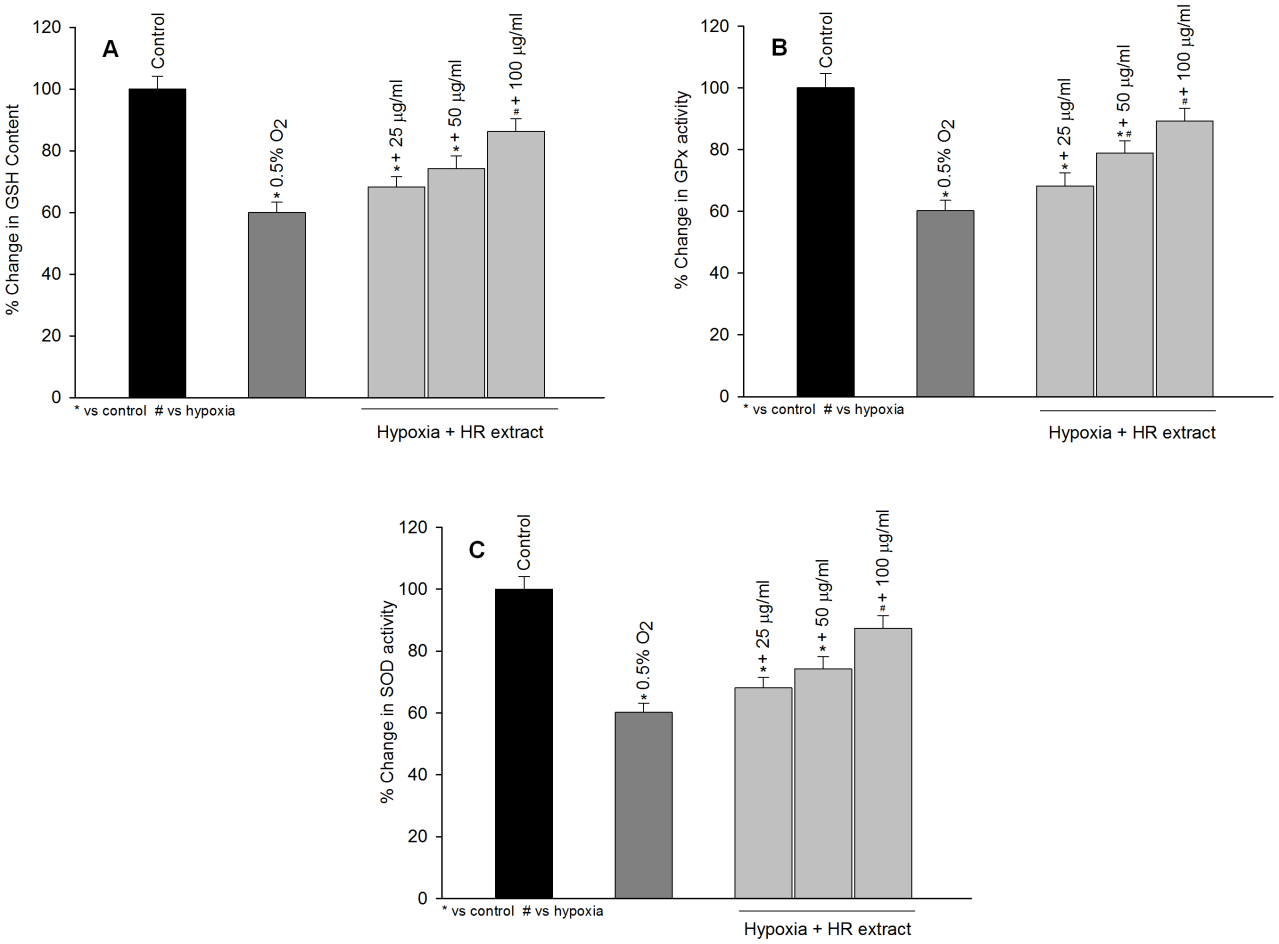
Protective efficacy of *Hippophae rhamnoides* extract on reduced glutathione levels (GSH; A), glutathione peroxidase (GPx; B) and superoxide dismutase (SOD) in HT22 cells following hypoxia exposure. The values are mean ± SE of three individual experiments. *p<0.05; * vs control; ^#^ vs hypoxia.

Figure 3 shows that exposure of HT22 cells to hypoxia resulted in a significant decrease in GSH, GPx and SOD levels in comparison to the unexposed cells (figure 3 A–C; dark gray bars). Treatment with the extract restored the cellular levels/activities of GSH, GPx and SOD which were compromised after hypoxia exposure and the protective effects were in dose dependent manner (figure 3 A–C; light gray bars).

### NFκB and Inflammatory Cytokines during Hypoxia Exposure in the Absence or Presence of Hippophae rhamnoides Extract

**Figure 4:**
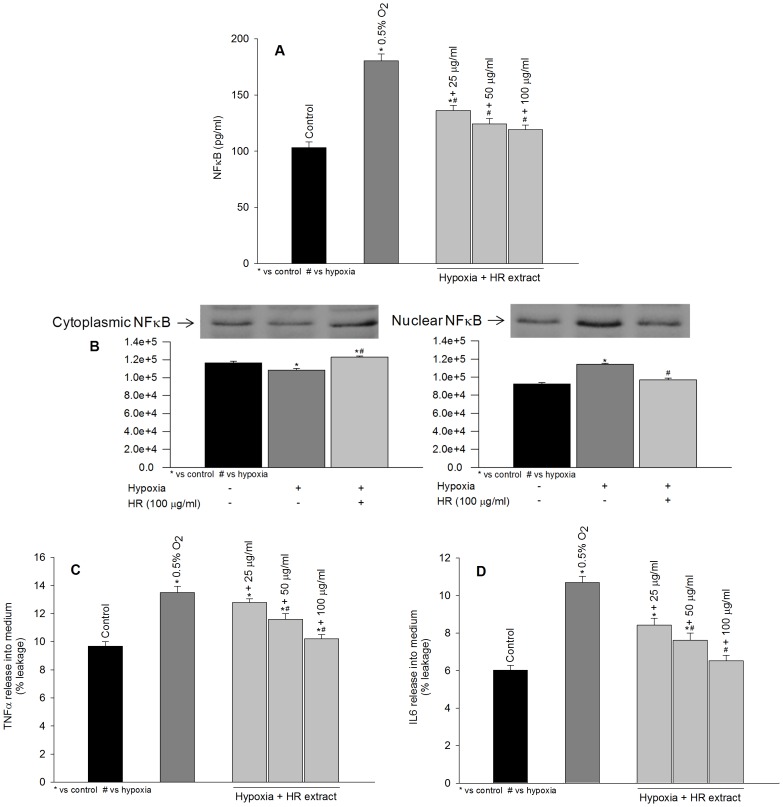
Effects of extract treatment on NFκB (A–B), TNFα (C) and IL6 (D) expression in HT22 cells following hypoxia exposure. *p<0.05; * vs control; ^#^ vs hypoxia.

**Figure 5:**
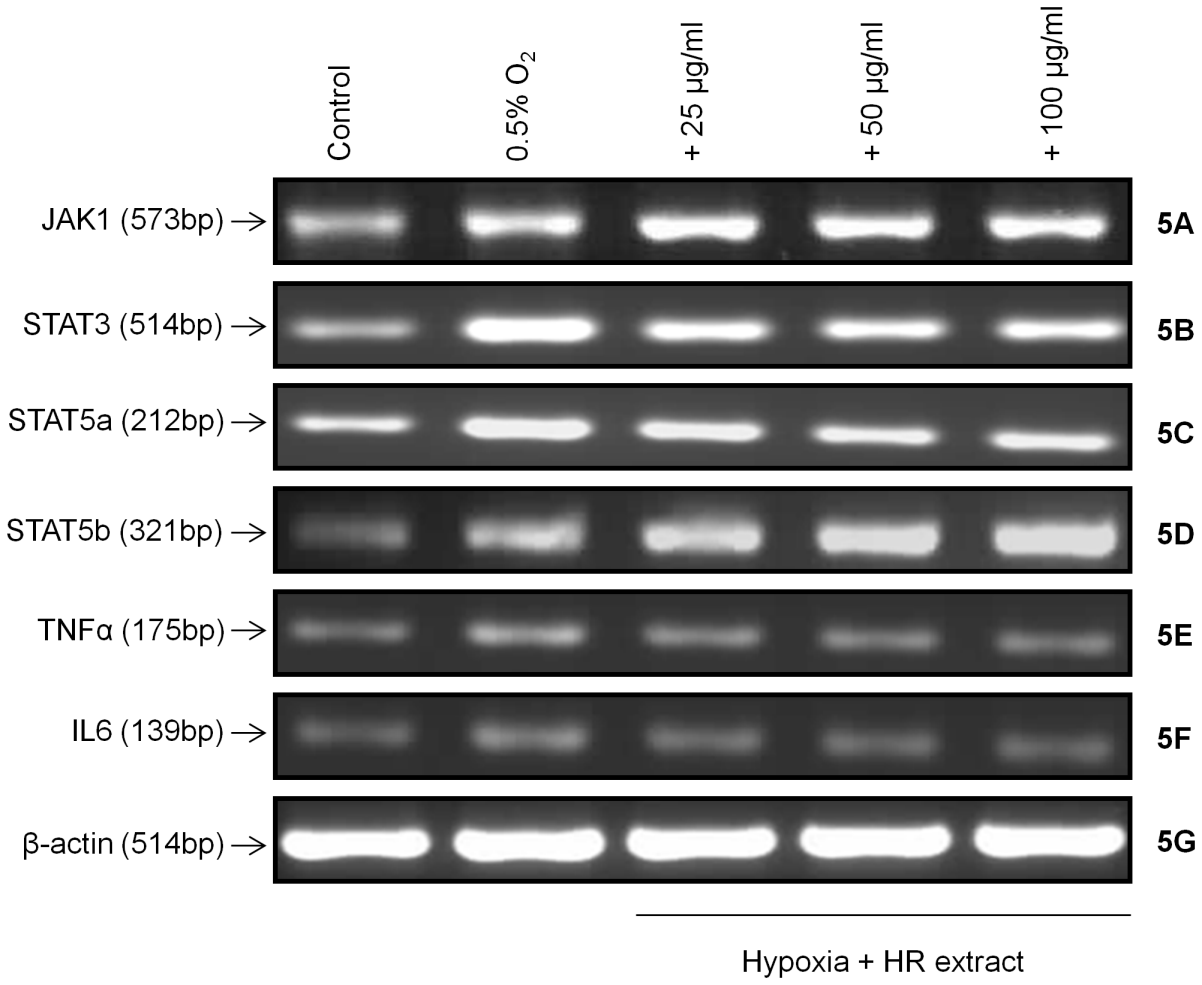
Immunoblot results of JAK1, STAT3, pSTAT3, STAT5, pSTAT5, STAT5a, STAT5b and β-actin in HT22 cells following hypoxia exposure with or without extract. *p<0.05; * vs control; ^#^ vs hypoxia.

Exposure of cells to hypoxia resulted in a marked increase in NFκB levels (figure 4A; dark gray bar) which is casually associated with the rise in reactive oxygen species. The treatment of these cells with extract attenuated such changes in NFκB induced by hypoxia probably by reducing reactive oxygen species load (figure 4A; light gray bars). To examine the translocation of NFκB into nucleus under hypoxia, cytoplasmic and nuclear fraction were prepared and assayed by Western blotting. Cells treated with hypoxia showed decreased expression of NFκB in cytoplasmic fraction (figure 4B, cytoplasmic; lane 2), with its increase in nuclear fraction (figure 4B, nuclear; lane 2) confirming its translocation into nucleus. The treatment of cells with *Hippophae rhamnoides* extract prevented NFκB translocation into nucleus observed following hypoxia exposure (figure 4B, cytoplasmic and nuclear; lane 3). Further, an increase in pro-inflammatory cytokines, TNFα and IL6, was recorded following hypoxia exposure relative to control cells (figure 4C–D; dark gray bars). *Hippophae rhamnoides* treatment attenuated hypoxia induced increase in TNFα and IL6 (figure 4C–D; light gray bars). Moreover, TNFα and IL6 mRNA were found to be higher in cells exposed to hypoxia in the absence of extract (figure 5E–F; lane 2). The cell treated with extract prevented such change in TNFα and IL6 mRNA induced by hypoxia (figure 5E–F; lanes 3–5).

### Janus Activated Kinase and Signal Transducers and Activators of Transcription during Hypoxia Exposure in the Absence or Presence of Hippophae rhamnoides Extract

**Figure 6:**
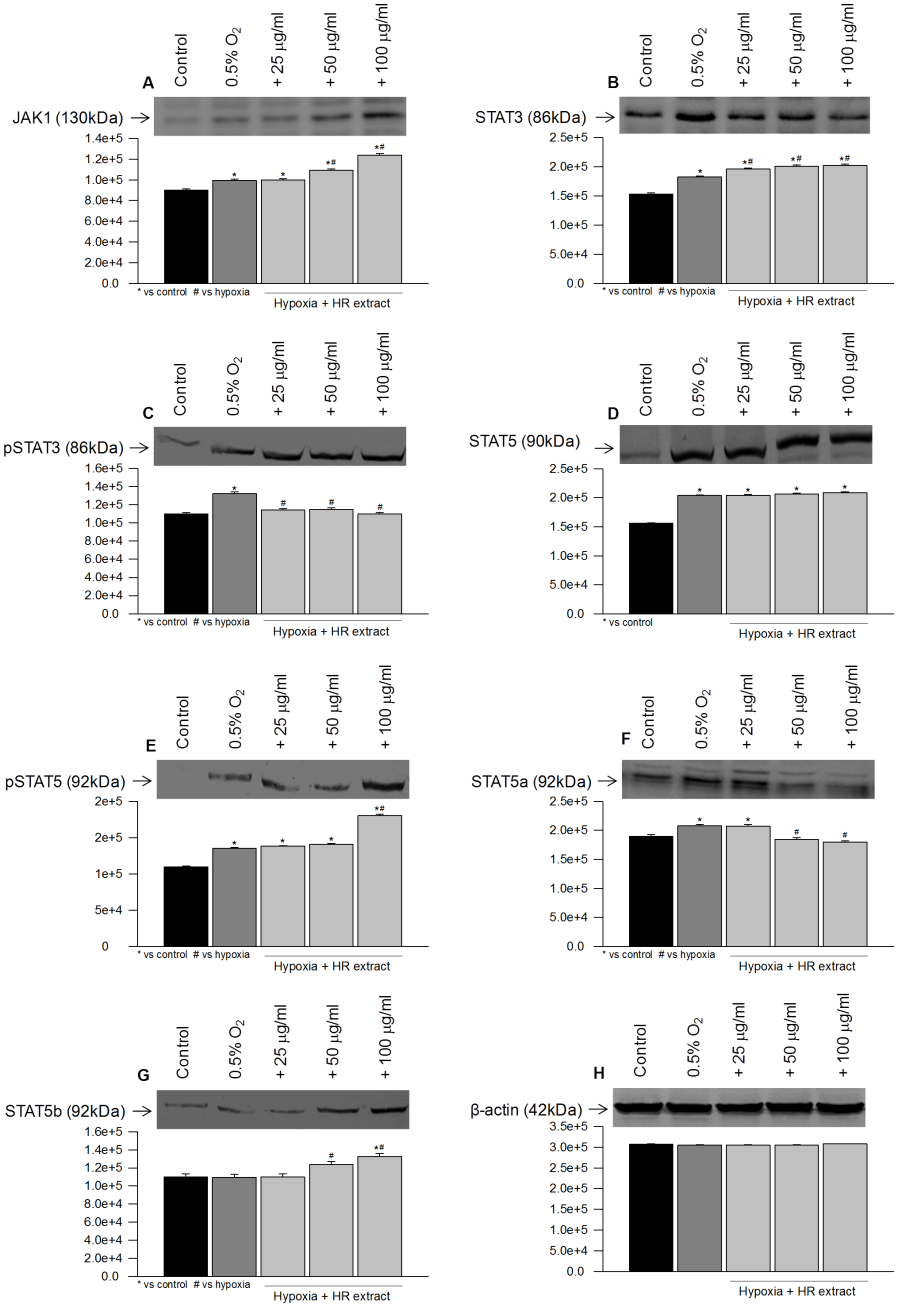
mRNA expressions of JAK1, STAT3, STAT5a, STAT5b, TNFα, IL6 and β-actin in HT22 cells following hypoxia exposure with or without extract. *p<0.05; * vs control; ^#^ vs hypoxia.

To examine activation of STAT proteins and dependence on upstream Janus activated kinases, HT22 cells were exposed to hypoxia and expression of JAK/STATs were measured by Western blotting. Exposure of cells to hypoxia resulted in an increased expression of JAK1, STAT3 and STAT5 (figure 6A–B, D; lane 2) which was concomitantly associated with phosphorylation of STAT3 and STAT5 (figure 6C, E; lane 2). An increase in mRNA levels of JAK1 and STAT3 were observed when cells were exposed to hypoxia (figure 5A–B; lane 2) in comparison to the controls (figure 5A–B; lane 1). STAT5a and STAT5b are reported to mediate signals for a broad spectrum of cytokines. In the present study, STAT5a was found to be highly expressive while no significant change was observed in STAT5b expression in cells exposed to hypoxia (figure 6F–G; lane 2). However, mRNA levels of STAT5a and STAT5b were found to be higher (figure 5C–D; lane 2) when compared with respective controls (figure 5C–D; lane 1).

Cells exposed to hypoxia in the presence of extract revealed augmented JAK1 and STAT5b expression (figure 6A, G; lanes 3–5) along with increased phosphorylated STAT5 (figure 6E; lanes 3–5). Similarly, mRNA expression of JAK1 and STAT5b were found to be enhanced in cells exposed to hypoxia in the presence of extract (figure 5A, D; lanes 3–5). Further, treatment of cells with extract decreased levels of STAT3 and STAT5a (figure 6B, F; lanes 3–5) in comparison to the cells exposed to the hypoxia in the absence of extract (figure 6B, F; lane 2). The mRNA levels of STAT3 and STAT5a were found to be reduced after extract treatment (figure 5B–C; lanes 3–5) in comparison to the hypoxic cells grown in the absence of extract (figure 5B–C; lane 2).

### Effects of JAK1, STAT3 and STAT5 Inhibitors on JAK1, pSTAT3, pSTAT5 Expression and Cell Viability under Hypoxia in the absence or Presence of Hippophae rhamnoides Extract

**Figure 7:**
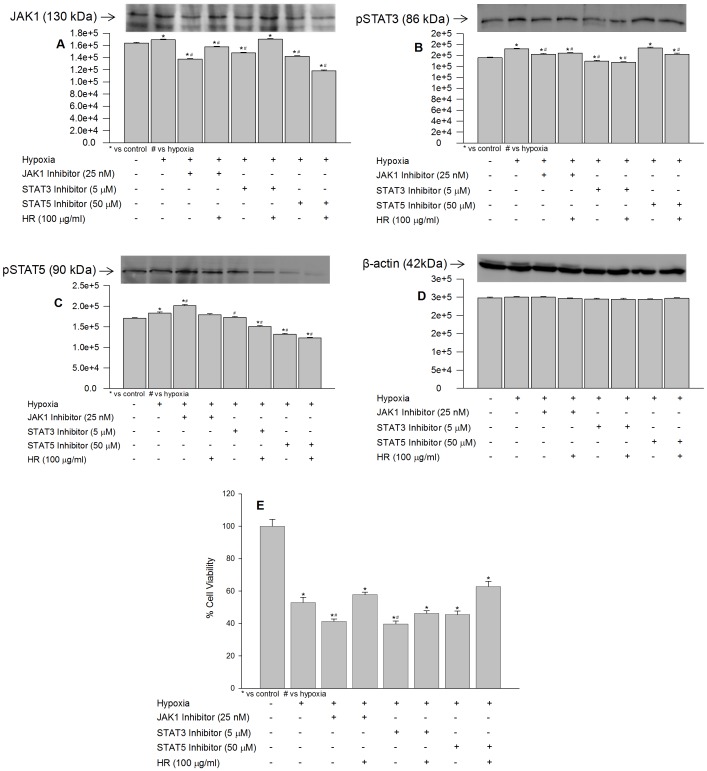
Effect of JAK1, STAT3 and STAT5 selective inhibitors on JAK1, pSTAT3, pSTAT5 expression and cell viability following hypoxia exposure with or without extract. *p<0.05; * vs control; ^#^ vs hypoxia.

The optimal concentration of JAK1, STAT3 and STAT5 specific inhibitors, that reduces protein expressions of JAK1, STAT3 and STAT5 maximally in HT22 cells, were found to be 25 nM, 5 µM and 50 µM, respectively, when exposed to normoxia or hypoxia (data not shown). Exposure of cells to hypoxia in the presence of specific inhibitor of JAK1 resulted in increased phosphorylation of STAT5 (figure 7C; lane 7) and decreased phosphorylation of STAT3 (figure 7B; lane 5) in comparison to hypoxia suggesting that in absence of functional JAK1 activation of STATs occur through alternate cytokine signaling. Cell treated STAT3 and STAT5 inhibitor, revealed decrease in Jak1 activity (figure 7A, lane 5, 7) in these cells indicating interdependence of STAT3 and STAT5 on JAK1 activity. Interestingly, hypoxic cells treated with JAK1 inhibitors in the presence of extract revealed higher expression of JAK1 comparing to the cells treated with JAK1 inhibitor alone, possibly extract did play role in restoring JAK1 activity in hypoxic cells (figure 7A; lane 4). Cells treated with extract and having suppressed STAT3 showed much higher JAK1 expression (figure 7A; lane 6). The findings suggest that extract augment JAK1 expression probably by decreasing STAT3 under hypoxia. Further, cells treated with extract and having suppressed STAT5 resulted in decreased JAK1 expression (figure 7A; lane 8) indicating phosphorylation of STAT5 was dependent on functionally active JAK1. Moreover, decrease in STAT3 was associated with higher STAT5 levels and vice versa indicating opposing role STAT3 to STAT5 in HT22 cells when exposed to hypoxia (figure 7B–C; lanes 5 and 7). The data suggest that *Hippophae rhamnoides* extract activated JAK1 activity in hypoxic cells and further changes in the levels of STATs were sequential.

In another study, the effects of JAK1, STAT3 and STAT5 inhibitors on cell survival under hypoxia in the absence or presence of *Hippophae rhamnoides* extract was studied. The cells exposed to hypoxia in presence of inhibitors of JAK1, STAT3 and STAT5 showed more cell death by 11.6%, 13.2% and 7.5%, respectively vs cell exposed to hypoxia without these inhibitors (figure 7E; bars 3, 5, 7 vs 2) suggesting that JAK/STAT components are essential for cell survival under hypoxia. Further, HT22 cells treated with these inhibitors in the presence of extract did not offer protection as that provided by the extract at 100 µg/ml concentration alone (figure 7E; bars 2, 4, 6, 8 and figure 2; bar 5). The data suggest that active cellular JAK1, STAT3 and STAT5 were required for the biological activity exhibited by the extract under hypoxia.

### Effects of JAK1, STAT3 and STAT5 Inhibitors on NFκB, IL6 and TNFα Expression under Hypoxia in the Absence or Presence of Hippophae rhamnoides Extract

**Figure 8:**
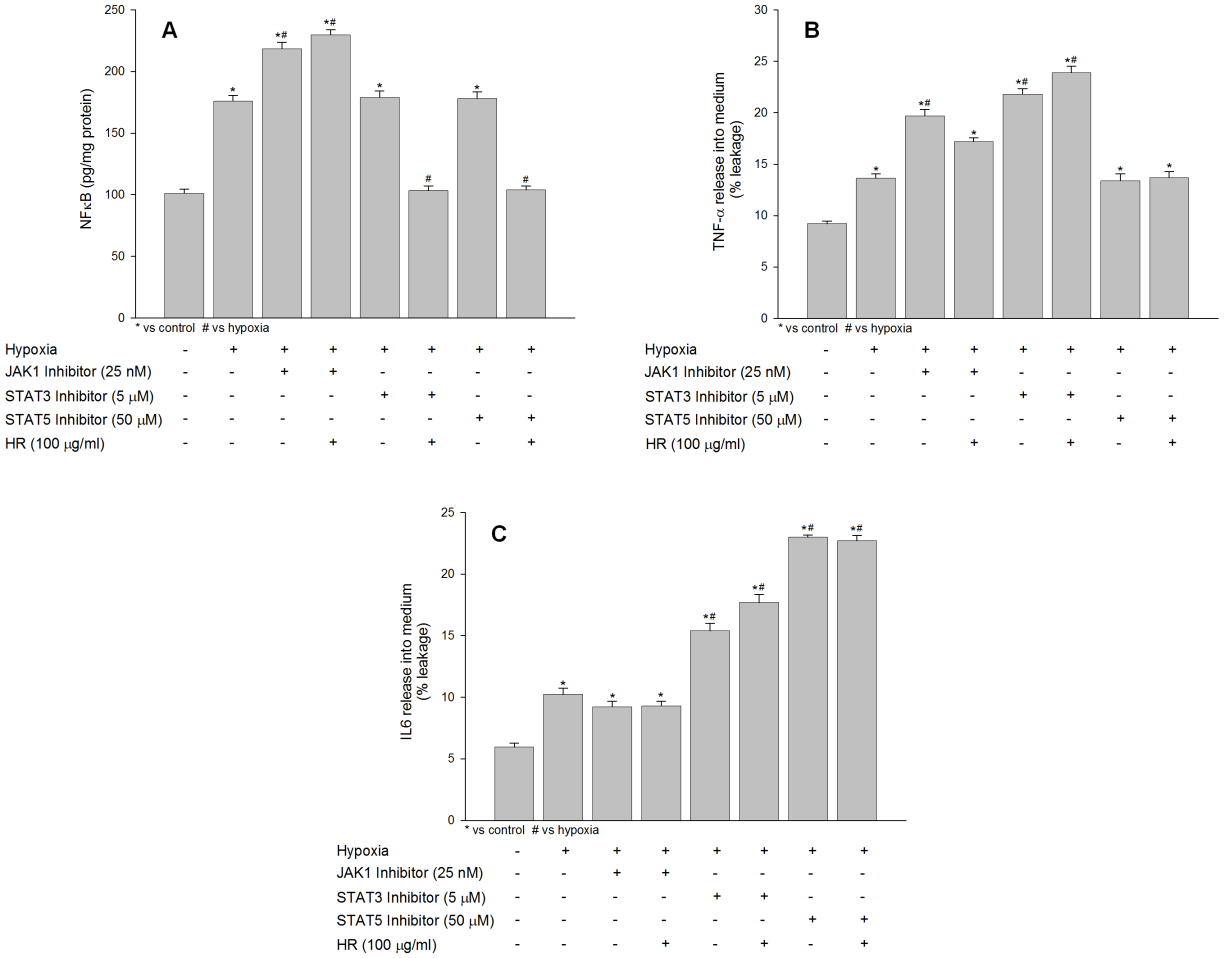
Effect of JAK1, STAT3 and STAT5 selective inhibitors on NFκB, IL6 and TNFα expression following hypoxia exposure with or without extract. *p<0.05; * vs control; ^#^ vs hypoxia.

The effect of JAK1, STAT3 and STAT5 inhibitors on hypoxia induced inflammatory response was studied in HT22 in the absence or presence of *Hippophae rhamnoides* extract using ELISA assay. The levels of NFκB were found to be increased in cell exposed to hypoxia in the presence of JAK1 inhibitor (figure 8A; bar 3). There was no change observed in NFκB expression in hypoxic cells grown in the presence of STAT3 and STAT5 inhibitor (figure 8A; bars 5, 7). Treatment of hypoxic cells with JAK1 inhibitors alone or in the presence of extract revealed higher expression of NFκB than the hypoxia cells evidencing cross talk between JAK1 and NFκB signaling (figure 8A; bar 4). Cells treated with extract and having suppressed STAT3 or STAT5 showed decreased levels NFκB comparing to the hypoxic cells (figure 8A; bars 6 and 8).

Exposure of cells to hypoxia in the presence of specific inhibitor of JAK1 resulted in increased release and accumulation of TNFα into culture medium (figure 8B; bar 3) when compared to hypoxic cells (figure 8B; bar 2) evidencing dependence of TNFα signaling on functional JAK1. The hypoxic cells treated with JAK1 inhibitor and extract revealed lesser release of TNFα into culture medium comparing to the cells treated with JAK1 inhibitor alone than hypoxic cells (figure 8B; bar 4) which might be due increased JAK1 activity in these cells by the extract treatment. TNFα which activates STAT3 was found to be accumulated in culture medium in cells treated with specific inhibitor of STAT3 (figure 8B; bar 5) when compared to hypoxic cells (figure 8B; bar 2). The accumulation of TNFα into culture medium further enhanced in cells treated with STAT3 inhibitor and extract in comparison to hypoxia exposed cells (figure 8B; bar 6 compared to bar 2) probably due to decrease in STAT3 levels by the extract. The cell treated with STAT5 inhibitor alone or with extract had no significant effect on TNFα release (figure 8B; bars 7, 8). This finding suggests that higher TNFα expression under low oxygen environment is responsible for maintaining higher cellular JAK/STATs activity thereby initiating pro-survival signaling pathway.

No significant change in IL6 release into culture medium in cells was observed in cells treated with JAK1 inhibitor alone or in the presence of extract (figure 8C; bars 3, 4) compared to hypoxic cells (figure 8C; bar 2). However, significant accumulation of IL6 was evident in cells treated with either STAT3 inhibitor or STAT5 inhibitor (figure 8C; bars 5, 7). IL6 can activate both STAT3 at lower concentration and STAT5 higher concentration in isolation or parallel and therefore suppression of any of component may lead into accumulation of IL6. The cells co-treated with STAT3 inhibitor and extract showed more accumulation of IL6 (figure 8C; bar 6) than the cells treated with STAT3 inhibitor alone (figure 8C; bar 5) which suggests that extract prevented hypoxia associated increase in STAT3 thereby causing accumulation of IL6 into culture medium. Further, there was no change observed in cell co-treated with STAT5 inhibitor and extract (figure 8C; bar 8) compared to the cell treated with STAT5 inhibitor alone (figure 8C; bar 7) suggesting STAT5 is not primary target of IL6 under low oxygen conditions.

### Effects of JAK1, STAT3 and STAT5 Inhibitors on Active Caspase 3 and Lactate Dehydrogenase Leakage under Hypoxia in the Absence or Presence of Hippophae rhamnoides Extract

**Figure 9:**
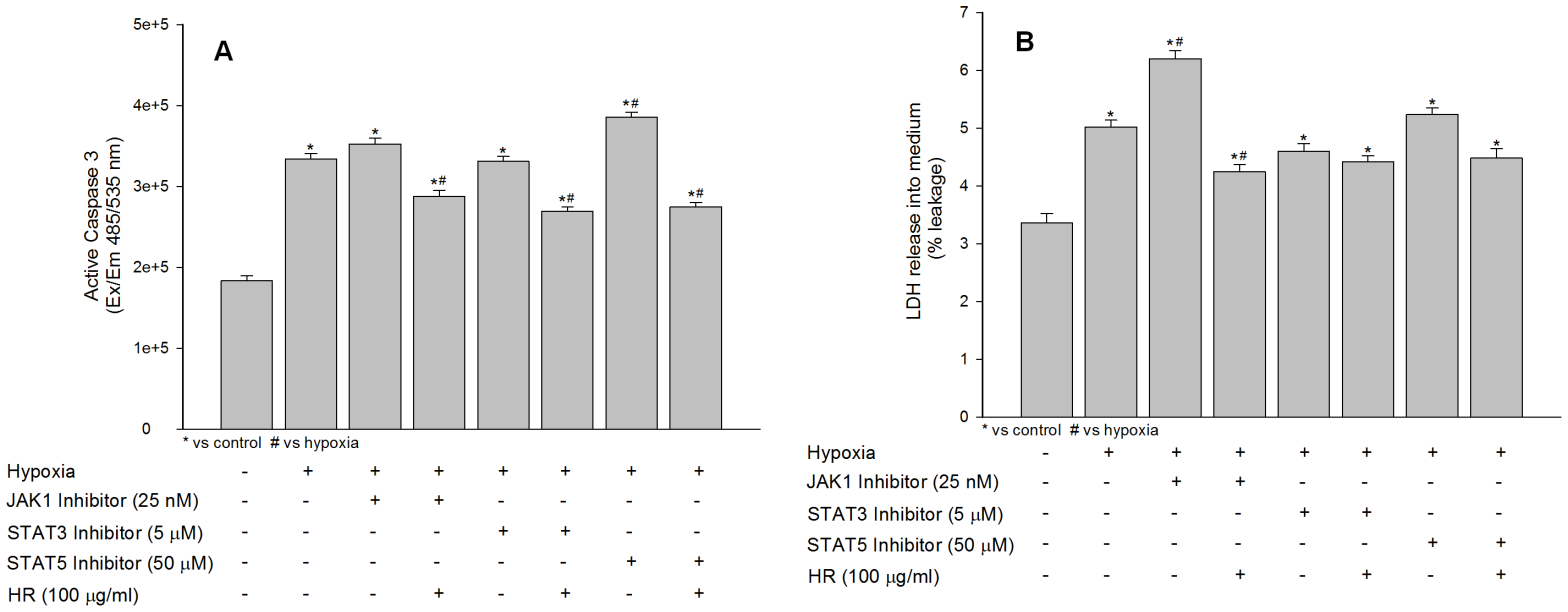
Effects of extract treatment on active caspase 3 activity (A) and lactate dehydrogenase leakage into culture medium (B) in HT22 cells following hypoxia exposure. *p<0.05; * vs control; ^#^ vs hypoxia.

The effect of JAK1, STAT3 and STAT5 inhibitors on hypoxia induced caspase 3 cleavage and lactate dehydrogenase release into medium was studied in HT22 in the absence or presence of *Hippophae rhamnoides* extract. The JAK/STAT signaling associated with various cellular events including cell survival, differentiation and death. To confirm whether JAK-STATs offer survival to HT22 cells under hypoxia, we measured levels of caspase 3 and lactate dehydrogenase leakage in cells suppressed of JAK1 or STAT3 or STAT5 in the absence or presence of *Hippophae rhamnoides* extract using specific inhibitors. The cells treated with JAK1 inhibitor showed statistically non significant increase in active caspase 3 compared to hypoxic cells which was accompanied by significantly leakage of lactate dehydrogenase into culture medium (figure 9A–B; bar 3) compared to hypoxic cells (figure 9A–B; bar 2) evidencing higher necrotic cell death in absence of functionally active JAK1. Further, cells co-treatment with JAK1 inhibitor and extract showed lesser expression of active caspase 3 and prevented leakage of lactate dehydrogenase into culture medium (figure 9A–B; bar 4) which may be due to augmented JAK1 expression by the extract. Cell treated with STAT3 inhibitor revealed reduced active caspase 3 and lactate dehydrogenase leakage into medium (figure 9A–B; bar 5) suggesting pro-apoptotic role of STAT3 in HT22 cells under low oxygen conditions. The treatment of cell with STAT3 inhibitor in the presence of extract further decreased active caspase 3 expression and lactate dehydrogenase leakage into medium compared to hypoxic cells (figure 9A–B; bar 6) indicating extract prevents hypoxia associated apoptosis by decreasing STAT3 activity in these cells. The cells treated with STAT5 inhibitor showed higher active caspase 3 levels (1.15 fold) and lactate dehydrogenase leakage (1.04 fold) into culture medium compared to hypoxic cells (figure 9A–B; bar 7) indicating STAT5 participates in preventing caspase 3 mediated apoptosis in HT22 cells under hypoxia environment. Co-treatment of cells with STAT5 inhibitor and extract revealed further decrease in active caspase 3 expression and prevented leakage of lactate dehydrogenase into culture medium (figure 9A–B; bar 8) which might be due to suppression of STAT3 and increased STAT5 expression by the extract.

## Discussion

In fact, many neuronal disorders involve oxidative stress and this may occur due to suppression of antioxidants. Several reports have shown that hypoxic stress induces reactive oxygen species production leading to cellular pathophysiology [14]. In the present study, HT22 cells exposed to hypoxia displayed higher reactive oxygen species with significant cell death. Further, cellular antioxidant status was found compromised after hypoxia exposure and treatment of *Hippophae rhamnoides* extract restored such changes which might be due to high phenolics and flavonoids content and many of the bioactive compounds possessed by the extract thereby preventing cell death and maintained antioxidant status. The leaves extract of *Hippophae rhamnoides* are reported to be rich in flavonoids [15] and is potent reactive oxygen species scavenger [16]. Cells subject to hypoxia revealed higher expression of active caspase 3 and leakage of lactate dehydrogenase into the culture medium confirming both apoptotic and necrotic cell death however, the cell death was predominantly apoptotic. It is reported that hypoxia exposure results in apoptosis in various cell types and is accompanied by apoptotic necrosis or secondary necrosis [17], [18], [19]. Treatment of cells with extract prevented activation of caspase 3 and lactate dehydrogenase leakage induced by the hypoxia and the effects were dose dependent.

TNFα is a pleiotropic and proinflammatory cytokine that has diverse cellular functions such as apoptosis, cell proliferation depending on the level and duration of the hypoxia exposure. Depending on the levels or duration of stressor, TNFα binds to cell surface receptors such as TNFR1 or TNFR2. A marked increase in TNFα release into culture medium was observed in hypoxic cells along with its transcriptional activation. It is documented that ratio of STATs in central nervous system play an important role in determining apoptotic or anti-apoptotic response [20] and various cytokines are involved in the regulation of the immune system and inflammation through JAK/STAT. In this study, it was observed that exposure of cells to hypoxia in the presence of specific inhibitor of JAK1 resulted in increased release and accumulation of TNFα into culture medium. The hypoxic cells treated with JAK1 inhibitor and extract decreased this TNFα release into culture medium. The cells treated with extract augments JAK1 activity both protein and mRNA levels. Therefore, an increase in JAK1 activity in these cells may have resulted in decreased TNFα release suggesting functional JAK1 is required for the tuning of TNFα. TNFα which activates STAT3 was found to be accumulated in culture medium in cells treated with specific inhibitor of STAT3 when compared to hypoxic cells. The accumulation of TNFα into culture medium further enhanced in cells co-treated with STAT3 inhibitor and extract in comparison to hypoxia exposed cells. The data suggests that extract prevented increase in STAT3 levels induced by hypoxia. Further, induction of JAK1 by the extract harmonizes TNFα to reduce neuronal cell death.

Growing evidence has indicated that cellular redox plays an essential role not only in cell survival, but also in cellular signaling pathways such as NFκB. It is well documented that treatment of cells with oxidants can activate the NFκB pathway [21]. A higher NFκB expression was evident in the cells exposed to hypoxia and treatment of *Hippophae rhamnoides* extract restored such changes possibly due to reduction in hypoxia-induced oxidative stress by the extract. A cross talk between JAK/STAT and NFκB pathways is well documented which could be mediated by various agents including suppressor of cytokine signaling [22], [23], [24]. In cells exposed to hypoxia in the presence of JAK1 inhibitor NFκB levels were found to be elevated however no change in its expression was observed with STAT3 or STAT5 suppression. It is reported that in JAK1 deficient cells, NFκB activation is observed without activation of STATs through TYK2 dependent pathway [25]. Further, it is also recorded that a strong link between oxidative stress and JAK/STAT signaling exist [26], [27]. The functional loss of JAK1, which regulate many cellular processes, may imbalance cellular homeostasis thereby activating NFκB through alternate channels to initiate survival response. It is documented that activation of cellular NFκB initiate survival response in many cell lines depending on the micro-environment and stimuli [28], [29]. The treatment of cells, lacking functional JAK1, with extract could not reduce NFκB evidencing cross talk between NFκB and JAK1 in HT22 cells under hypoxic micro environment. In cell having suppressed STAT3 or STAT5, treatment with extract reduced NFκB expression indicating changes in NFκB levels under hypoxia is independent of phosphorylation STAT3 or STAT5.

IL6 belongs to a family of cytokines and plays a crucial role in acute inflammatory response, growth and survival signaling. IL6 binds to IL6Rα receptor leading in activation of Janus activated kinase and phosphorylation of signal transducer and activator of transcription (STAT) family members. JAK/STAT signaling plays an important role in executing downstream effects of various cytokines induced by various stress response mechanisms including hypoxia thereby exerting many physiological actions such as cell survival, differentiating, death etc. depending on the levels of exposure and cell type. There are reports stating activation of JAK/STATs by hypoxia [8], [30] and further downstream effect of IL6 is executed through JAK/STAT pathway [31]. Particularly, in central nervous system dysregulation of JAK/STAT signaling is associated with brain inflammation process and many neuronal disorders [32]. In the present study, an increase in expression of JAK1 on exposure to hypoxia was noticed with phosphorylation of STAT3 and STAT5. Our results are in accordance with previous reports showing increase in mRNA levels of JAK1, STAT3 and STAT5 on exposure to hypoxia [8], [33]. Activation of STAT3 via IL6 [34], [35], [36] is associated with the angiogenesis process in many cell types under hypoxia exposure [37], [38], [39]. It is documented that IL6 is pleiotrophic cytokine activating multiple pathways including JAK/STAT3 but at higher concentrations it also activate STAT5 to establish a negative feedback loop to balance STAT3 dependent inflammatory response [40]. In the present study, a significant accumulation of IL6 was observed in cells treated having suppressed STAT3. Further, cells co-treated with STAT3 inhibitor and extract showed excessive accumulation of IL6 than the cells treated with STAT3 inhibitor alone which confirm that extract prevented hypoxia associated increase in STAT3 evidencing interdependence of IL6 and STAT3 under hypoxic environment. Further, accumulation of IL6 was evident in cells treated with STAT5 inhibitor confirming cross talk between IL6 and STAT5 as well. However, no change was observed in cell co-treated with STAT5 inhibitor and extract suggesting STAT5 is not primary target of IL6 under low oxygen conditions. Although, the basal levels of the STAT3 proteins were lowered with the extract treatment, the phosphorylation of STAT3 was evident which was independent of IL6 activation. It is documented that extracts from various plants induce growth factors, angiogenesis and extracellular matrix proteins [41]. Therefore, it is possible that *Hippophae rhamnoides* promoted growth factors signaling which phosphorylated STAT3 independent of IL6. STAT3 is important in transducing pro-apoptotic signals and along with STAT5 promotes cell survival [32], [42]. In this study, it was observed that suppression of STAT3 decreased active caspase 3 and lactate dehydrogenase leakage into culture medium compared to hypoxia and when STAT5 is inhibited a reversed trend was visible. However, suppression of STAT3 or STAT5 results in more cell death than hypoxic cells evidencing necessity of functional STAT3 and STAT5 for cell survival in low oxygen environment. The data obtained from the study indicate that a balance between STAT3 and STAT5 in HT22 cell under low hypoxia is crucial for cell fate.

Further, STAT5a and STAT5b mediate signals for a broad spectrum of cytokines. In this study, activation of JAK1 was observed accompanied by phosphorylation of STAT3, STAT5a and STAT5b following hypoxia exposure both at protein level and their up-regulations at mRNA levels. It is documented that JAK1 activation is accompanied by the STAT3 and STAT5b phosphorylation in human B cells [34]. It is also reported that phosphorylation of STAT3 is dependent on upregulation of Akt in response to IL10 but independent of Jak1 in cardiomyocyte [35]. The treatment of cells with the extract resulted in upregulation of JAK1 and STAT5b with the phosphorylation of STAT3 and STAT5. Moreover, the up-regulation of IL6, STAT3, STAT5a mRNA levels in cells exposed to hypoxia was minimized after the extract treatment. The findings indicate that extract treatment regulated JAK1/STAT5b in HT22 cells exposed to hypoxia. The JAK1/STAT5b induction after *Hippophae rhamnoides* extract treatment probably fine tune IL6/STAT3/STAT5a mediated responses to hypoxia thereby regulating angiogenesis and erythropoeisis processes.

Overall, the present study highlights that *Hippophae rhamnoides* extract treatment renders neuroprotection against hypoxia in HT22 cells via induction of JAK1/STAT5b pathway.

## Materials and Methods

### Apparatus

ASE 350 Dionex Corporation (Sunnyvale, CA, USA), Spectrophotometer Bio-Rad. ELISA reader (Molecular Devices, USA), Spectrofluorimeter (Varian, USA).

### Reagents

Dulbecco’s modified Eagle’s medium (DMEM) and fetal bovine serum (FBS) were purchased from Invitrogen (Carlsbad, CA, USA). Specific antibodies against JAK1, STAT3, pSTAT3, STAT5 and pSTAT5 and HRP conjugated secondary antibodies were purchased from Santa Cruz Biotechnology Inc., California, USA while NFκB p65 and β-actin antibodies were procured from Abcam Inc, Cambridge, MA, USA. NFκB, TNFα and IL6 Elisa kits were purchased from (R & D Systems, USA). Lowry and Bradford protein assay reagents were purchased from Pierce (Rockford, IL, USA) and St. Louis, MO, USA), respectively. Selective JAK1 inhibitor (Cat no. 420099), STAT3 inhibitor III WP 1066 (Cat no. 573097) and STAT5 inhibitor (Cat no. 573108) was purchased from Merck KGaA, Darmstadt, Germany. Other high quality research grade chemicals were purchased from Sigma (St. Louis, MO, USA).

### Plant Material

*Hippophae rhamnoides* leaves were collected in the month of September from hilly regions of Western Himalayas, India, where the plant grows widely under natural conditions. The Defence Institute of High Altitude Research, Leh, India, where the voucher specimen (DIP-HIP/2012) of the plant material is preserved in the herbarium, carried out the ethanobotanical identification of the plant. Fresh leaves of seabuckthorn were cleaned and washed thoroughly with water and re-washed with distilled water. Washed fresh leaves were dried under shade in a clean, dust free environment and crushed using laboratory blender. The permission for the collection of study material was obtained from Defence Institute of High Altitude Research, Ministry of Defence, Government of India. The field study did not involve endangered or protected species.

### Extract Preparation

Aqueous lyophilized extract of *Hippophae rhamnoides* dried leaves was prepared by maceration method as described earlier [43]. One gram of dried *Hippophae rhamnoides* leaves produced 0.2 g of lyophilized seabuckthorn aqueous extract powder.

### Determination of Total Phenol Content

The total phenolic content in extract was estimated using Folin–Ciocalteu reagent (FCR) based assay [44]. The following were added to a tube; 20 µl of stock solution (1 mg/ml) of the extract, 80 µl of water and 500 µl of FCR. After 5 min incubation in the dark at room temperature, 400 µl of 7.5% sodium carbonate solution was added. The mixture was incubated in the dark for 30 min at room temperature and absorbance of the color developed was read at 765 nm**.** Total phenols (mg/g) in the extract were expressed as gallic acid equivalent (GAE), using standard curve prepared from gallic acid (0.1 mg/ml) solution.

### Determination of Total Flavonoid Content

Total flavonoid content was estimated by the aluminum chloride colorimetric assay as described elsewhere [45] using rutin as a standard. 1 ml of extract (1 mg/ml) was added to 4 ml distilled water and subsequently 0.3 ml of 5% NaNO_2_ solution was added. After 5 min, 0.3 ml of 10% AlCl_3_ solution was added and allowed to stand for 5 min, then 0.2 ml of 4% NaOH solution was added to the mixture and the volume was adjusted up to 10 ml with distilled water. Absorbance of the mixture was read at 510 nm. Total flavonoid content (mg/g) in the extract was expressed as rutin equivalent.

### FRAP (Ferric Reducing Antioxidant Power) Assay

The FRAP assay was estimated using method described elsewhere [46]. The fresh FRAP working solution was prepared by mixing 25 ml of 300mM acetate buffer (3.1 g C_2_H_3_NaO_2_ 3 H_2_O and 16 ml C_2_H_4_O_2_, pH 3.6), 2.5 ml of 10 mM TPTZ (2, 4, 6- tripyridyl-s-triazine in 40 mM HCl), and 2.5 ml of 20 mM FeCl_3_.6 H_2_O solution which was warmed at 37°C prior to use. 150 µl of extract (1 mg/ml) was allowed to react with 2850 µl of the FRAP solution for 30 min in dark and the absorbance of the color developed was read at 593 nm. Results are expressed as mg of Trolox equivalent/g of extract, using standard curve prepared from Trolox solution.

### HPLC Fingerprinting

HPLC analysis of the subcritical water extract of *Hippophae rhamnoides* was performed using Waters HPLC system (Waters Corporation, USA) equipped with Waters 515 HPLC pump, Waters 717 plus autosampler and Waters 2487 UV detector. Separation was performed in a symmetry C18 250 mm×4.7 mm ID; 5 µm column (Waters, USA) by maintaining the isocratic flow rate (1 ml/min) of the mobile phase consisting of 1% acetic acid in water (A) methanol (B), applied as gradient elution. The standard substance isorhamnetin and kaempferol were analyzed in the same conditions. Peaks were assigned by spiking the samples with standard substance and comparison of the retention times and spectral matching.

### Cells and Culture Conditions

The cell line HT22 is a subclone of the HT4 hippocampal cell line [47]. HT22 cells (kind gift of Dr. Dave Schubert, Salk Institute, San Diego, CA, USA), a murine immortalized hippocampal neuronal cell line was maintained in DMEM medium containing 10% FBS and 1X antibiotic-antimycotic solution as described elsewhere [48]. Cells were then plated in a density of 1×10^4^ cells/ml in 96 well plates or 1×10^6^ cells/ml in 60 mm plates or 3×10^6^ cells/ml in 100 mm plates and maintained for 24 h following treatments. Untreated cells were cultured in normoxia incubator (Heracell 150i, Thermo Scientific, USA) maintaining 5% CO_2_ at 37°C in DMEM medium while hypoxic conditions were achieved by culturing cells in an hypoxia incubator (Jouan, Saint-Nazaire, France) maintained at 37°C, 0.5% O_2_, 5% CO_2_, and 94% N_2_ atmosphere. Viability of the cells was determined by MTT assay. Cells were exposed to 0.5% O_2_ for 24 h in presence or absence of *Hippophae rhamnoides* extract.

### MTT (Methylthiazole Tetrazolium) Cytotoxicity Assay

The MTT assay, which is based on conversion of yellow tetrazolium salt to purple-formazan crystals by metabolically active cells, provides a quantitative determination of viable cells. Cells seeded at the density of 1×10^4^ cells per well in 96 well tissue culture plates were allowed to adhere for 24 h at 37°C. Cells were then treated with various concentration of *Hippophae rhamnoides extract* dissolved in DMEM media. Cells were exposed to normoxia and hypoxia for 24 h. Cytotoxicity was assessed by MTT assay. 50 µl of MTT (1 mg/ml) was added to each well and incubated for 4 h at 37°C. Formazan crystals were solubilized in 100 µl of DMSO by incubating in shaking condition at room temperature for 5 min. Absorbance was taken at 570 nm with 630 nm as reference filter. Absorbance given by untreated cells was taken as 100% cell survival.

### Assay for Intracellular Redox State

Intracellular redox state levels were measured using the fluorescent dye 2,7-dichlorofluorescein diacetate (H2-DCFH-DA). Briefly, cells were washed once with HBSS and incubated in the same buffer containing 5–10 µg of DCFH-DA for 30 min at 37°C. Intracellular fluorescence was detected with excitation at 485 nm and emission at 530 nm using Spectra Max Gemini EM (Molecular Devices, Sunnyvale, CA).

### Biochemical Analysis

After normoxic and hypoxic exposure, the cells were harvested by trypsinization, washed and sonicated for 10 sec in sterile phosphate buffered saline (PBS; pH 7.4) and then centrifuged (Sigma, Munich, Germany) at 1500 *g* for 10 min at 4°C. The pellet containing cell debris was discarded and the supernatant was used to determine GSH, GPx and SOD levels using commercial diagnostic kits (Randox, UK). The protein content in the homogenate was determined by the Lowry method as mentioned elsewhere [49].

### Lactate Dehydrogenase (LDH) Assay

After normoxic and hypoxic exposure, the culture medium was collected for the estimation of extracellular lactate dehydrogenase. Then the cells were harvested by trypsinization, washed and sonicated for 10 sec in sterile phosphate buffered saline (PBS; pH 7.4) and then centrifuged (Sigma, Munich, Germany) at 1500 *g* for 10 min at 4°C. The pellet containing cell debris was discarded and the supernatant was used to measure intracellular lactate dehydrogenase. The extra cellular and intra cellular lactate dehydrogenase was measured using commercial diagnostic kit (Randox, UK). Leakage of intracellular enzymes was expressed as percent leakage.

### Caspase 3 Assay

After normoxic and hypoxic exposure, cells were analyzed for active Caspase 3 using Caspase 3 fluorometric proteases assay kit (Biovision, USA). Briefly, cells were washed sterile phosphate buffered saline (PBS; pH 7.4), resupended of chilled cell lysis buffer and incubated on ice for 10 minutes. Then a 50 µl of 2X reaction buffer (containing 10 mM DTT) was added followed by addition of 5 µl of the 1 mM DEVD-AFC substrate and incubate mixture at 37°C for 1 hour. The cleavage of substrate was detected at excitation 485 nm and emission 535 nm using Spectra Max Gemini EM (Molecular Devices, Sunnyvale, CA).

### Cellular Protein Preparation, Western Blotting and Quantitative ELISA

Following indicated treatments, cells were washed thrice with ice-cold phosphate buffered saline (PBS) and lysed in ice-cold cell lysis buffer (50 mM Tris-HCl, pH 7.5, with 120 mM NaCl, 10 mM sodium fluoride, 10 mM sodium pyrophosphate, 2 mM EDTA, 1 mM sodium orthovanadate, 1 mM phenylmethylsulfonyl fluoride, 1% NP-40 and protease inhibitor cocktail). Then cell lysates were centrifuged at 10,000 rpm for 30 minutes. The pellet containing tissue/cell debris was discarded and the supernatant was used for the further analysis. The samples were appropriately diluted for the estimation of protein using Bradford reagent (Sigma, St. Louis, MO).

Quantitative ELISA kits were used for studying expression of NFκB, TNFα and IL6 following the manufactures instructions (R&D Systems, USA). In general, equalized amounts of protein samples were loaded onto each specific antibody coated wells and incubated. After incubation the ELISA plates were further incubated with horseradish peroxidase-conjugated secondary antibodies following washing. The color was generated by incubating the ELISA wells with TMB substrate and color generation was terminated by stop solution. The yellow color generated for the samples was read at 450 nm against the specific standards provided in the kit. The extracellular and intracellular levels of TNFα and IL6 were measured and data is presented as % leakage.

Western blot analysis was used for the detection of JAK/STATs. Briefly, 40 µg of protein was resolved on 10–12% SDS-polyacrylamide gel which was subsequently transferred onto PVDF membrane. The membranes were probed with respective primary antibodies; JAK1 (1∶200), STAT3 (1∶200), p-STAT3 (1∶200), STAT5 (1∶200), p-STAT5 (1∶200), NFκB (1∶1000), β-actin (1∶2000) followed by HRP conjugated secondary antibodies. Immunoblots were detected by 3, 3′-Diaminobenzidine (DAB). To examine nuclear translocation of NFκB, cytoplasmic and nuclear fractions were prepared using cell and nuclear lysis kit (NXTRACT, Sigma, St. Louis, MO). The subcellular fractions were then assayed by Western blot.

### cDNA Synthesis and Reverse Transcription-polymerase Chain Reaction Analysis

Hippocampal neurons were lysed and total RNA was extracted with the TRIZOL reagent. Total RNA was qualified and quantified at 260 nm and 280 nm using Beckman spectrometry (USA). The cDNA was synthesized in a 20 µl reaction mixture, according to the protocol of the manufacturer (Thermo Scientific, USA). Specific cDNA was amplified by 35 PCR cycles at 94°C for 1 min, 55–65°C for 1 min and 72°C for 2 min. The PCR primers used in the study are shown in Table 1.

**Table 1:** PCR primer sequences and annealing temperature.

Gene	Upper Sequence	Lower Sequence	Product(bps)	Temperature(°C)	Reference
JAK1	CGGAACCAATGACAACGAACAGTC	CCAAGGTAGCCAGGTATTTCACC	473	63	[50]
STAT3	CTTGGGCATCAATCCTGTGG	TGCTGCTTGGTGTATGGCTCTAC	514	63	[50]
STAT5a	ATTACACTCCTGTACTTGCGA	GGTCAAACTCGCCATCTTGG	212	58.4	[51]
STAT5b	TCCCCTGTGAGCCCGCAAC	GGTGAGGTCTGGTCATGACT	321	62	[51]
TNFα	CATCTTCTCAAAATTCGAGTGACAA	TGGGAGTAGACAAGGTACAACCC	175	45	[52]
IL6	ATGGATGCTACCAAACTGGAT	TGAAGGACTCTGGCTTTGTCT	139	45	[52]
Actin	TGTGATGGTGGGAATGGGTCAG	TTTGATGTCACGCACGATTTCC	514	58	[53]

### Treatment of HT22 Cells with Selective Inhibitors of JAK1, STAT3 and STAT5

For treatment with JAK1, STAT3 and STAT5 inhibitors, the stock solutions were made in DMSO and diluted in cell culture medium to make desired concentrations before treatment. An equal amount of DMSO was added to normoxia and hypoxia cells nullifying its effect in the experiment. The cells were grown in 96-well plates for the cell viability experiments and treated with various concentrations of selective inhibitors of JAK1, STAT3 and STAT5. Then the cells were kept in normoxic and hypoxic conditions as mentioned earlier in cells and culture conditions section for 24 h and at the end of experimental period cell viability was determined by MTT assay. In another experiments, cells were grown in 100 mm cell culture plates under normoxia and hypoxia conditions in the presence of selective inhibitors of JAK1, STAT3, STAT5 and the extract and at the end of experimental duration cell lysate was prepared for the protein expression studies using Western analysis and Elisa.

### Data Analysis

All the experiments were performed on three different occasions and data are presented as mean ± SE. The data was analyzed by one-way ANOVA followed by Dunnet’s test for comparing control or hypoxia and the various groups, using GraphPad software. Statistical significance was estimated at the 5% level.
